# Considerations of AOX Functionality Revealed by Critical Motifs and Unique Domains

**DOI:** 10.3390/ijms19102972

**Published:** 2018-09-29

**Authors:** Rhoda A. T. Brew-Appiah, Karen A. Sanguinet

**Affiliations:** Department of Crop and Soil Sciences, Washington State University, Pullman, WA 99164-6420, USA

**Keywords:** environmental stress, wheat, barley, rice, *Trypanosoma brucei*

## Abstract

An understanding of the genes and mechanisms regulating environmental stress in crops is critical for boosting agricultural yield and safeguarding food security. Under adverse conditions, response pathways are activated for tolerance or resistance. In multiple species, the alternative oxidase (*AOX*) genes encode proteins which help in this process. Recently, this gene family has been extensively investigated in the vital crop plants, wheat, barley and rice. Cumulatively, these three species and/or their wild ancestors contain the genes for *AOX1a*, *AOX1c*, *AOX1e*, and *AOX1d*, and common patterns in the protein isoforms have been documented. Here, we add more information on these trends by emphasizing motifs that could affect expression, and by utilizing the most recent discoveries from the AOX isoform in *Trypanosoma brucei* to highlight clade-dependent biases. The new perspectives may have implications on how the *AOX* gene family has evolved and functions in monocots. The common or divergent amino acid substitutions between these grasses and the parasite are noted, and the potential effects of these changes are discussed. There is the hope that the insights gained will inform the way future *AOX* research is performed in monocots, in order to optimize crop production for food, feed, and fuel.

## 1. Introduction

Barley was domesticated over 10,000 years ago, and is one of four major cereal crops accounting for a third of annual global food production [1,2]. It is adaptive to a range of geographic conditions and is used mostly for food and feed [3,4]. In addition, it has a sequenced genome, partly due to the availability of ample diverse germplasm, as well as its potential as a model for the close relative wheat [4]. Given its wide climatic range, barley may be studied for increased food security and for stress tolerance, the outcomes of which can be extrapolated to the more recalcitrant polyploid relative wheat [5,6]. Rice is another monocot that is highly produced and consumed around the world [1]. The sequenced genomes and transcriptomes, as well as substantial germplasm bank, have allowed for extensive study of this cereal for the improvement of drought tolerance and the generation of new varieties for sustained or enhanced agricultural production [7,8,9].

One of the genes implicated in stress tolerance in multiple organisms is alternative oxidase *(AOX)* [10,11]. This gene family is responsive to stress in hexaploid wheat ([12] and references therein) as well as in barley and rice [13,14]. Phylogenetic analysis has shown the barley *AOX* (*HvAOX*) family is closely related to the larger *AOX* gene family in wheat [12] and, thus, exploring the barley family in order to better understand wheat is a viable endeavor. Similar work has also shown that the rice *AOX* family (*OsAOX*) contains the *AOX1e* clade present in wheat, but absent in barley [12,14]. The *AOX* gene structures, as well as expression patterns have been elucidated in the most recent work in both barley and rice. The protein sequences and the implications for structure and function are also discussed [14]. The current study aims to provide a different perspective on the known gene and isoform sequences in barley, rice, and wheat, which provides new avenues for the future developmental and functional characterization of the *AOX* gene and protein families in cereals. This alternate view increases our understanding of this gene family in the grasses, and may be utilized in innovative research of more grass species in the effort to boost crop production to meet global food and fuel needs.

## 2. Alternative Oxidase (*AOX*) Regulation in Monocots

While it has been previously shown that *HvAOX* and *OsAOX* expression is responsive to stresses and hormones [13,14], their expression levels could also be induced by other factors. There are several positive and negative AOX regulators known to affect expression in the dicot *Arabidopsis thaliana*. These have been discovered via hormone/chemical applications, mutant screens, and genetic studies, and classified as positive or negative, based on the activation/derepression or inhibition of *AOX* expression ([15,16,17] and references therein). The motifs of a selection of these regulators were found and summarized in wheat [12]. Binding sites for some of these regulators are also present in the *HvAOX* promoter regions (defined as 1500 bp upstream of translation start site). Using available search algorithms [18,19], we observe binding sites for the positive regulators ANAC013 (*HvAOX1a*, *HvAOX1c*), ANAC017 (*HvAOX1a*, *HvAOX1c*), ANAC053 (*HvAOX1a*), ANAC078 (*HvAOX1a*), AtWRKY63 (*HvAOX1d2*), and for the negative regulator ABI4 (*HvAOX1a*, *HvAOX1d1*, *HvAOX1d2*) (Table 1 and Table S1), supporting the experimental results for the response of *HvAOX* to abscisic acid. Binding sites for positive regulators are also found in rice; ANAC013 (*OsAOX1a*), ANAC017 (*OsAOX1a*), ANAC053 (*OsAOX1a*), and ANAC078 (*OsAOX1e*). The exploration of these regulators in monocots is lacking, and the interaction between these regulators and other synergistic or antagonistic pathways is yet to be clarified.

The presence of motifs for ANAC013 may indicate specific modes of control, such as mitochondrial retrograde regulation (MRR), a possibility that has also been observed in wheat [12,17]. MRR refers to the signaling that occurs between the mitochondria and the nucleus. The majority of research on this very complex process has focused on the identification and interactions of the molecular components involved [20]. An observation that has emerged is that MRR is central to stress tolerance [21,22,23]. In *A. thaliana*, a specific number of genes are highly responsive to MRR perturbation reagents. This group of genes, which includes *AOX*, has been dubbed the *MITOCHONDRIAL DYSFUNCTION STIMULON* (*MDS*) genes [17]. *MDS* genes are regulated via the binding of ANAC013 to the motif CTTGNNNNNCAMG, known as the mitochondrial dysfunction motif (MDM). This motif is present in the promoter regions of *HvAOX1a*, *HvAOX1c*, and *OsAOX1a* (Table 1 and Table S1). Some of these *MDS* genes may possess a variant of the MDM with a single nucleotide deviation. A search with the sequence YTTGNNNNNVAMV, covering all permutations previously reported [17], shows that *HvAOX1a*, *HvAOX1d1*, *OsAOX1a*, and *OsAOX1e* have the MDM with a single nucleotide deviation (Table 1 and Table S1). MRR is, therefore, plausible for *HvAOX1d1* and *OsAOX1e*, which lack the stringent MDM. As this MDM and its derivatives have also been found in wheat [12], these observations give insight into putative developmental pathways involving *AOX* in monocots, and allows for the possibility of studying the similarities and differences in *AOX* expression in monocots and dicots in the context of MRR. It also encourages exploration within the monocots in order to determine how MRR regulation has diverged within clades and between different species.

## 3. Probing AOX Function: Is Past Performance an Indicator of Future Failure?

**Figure 1:**
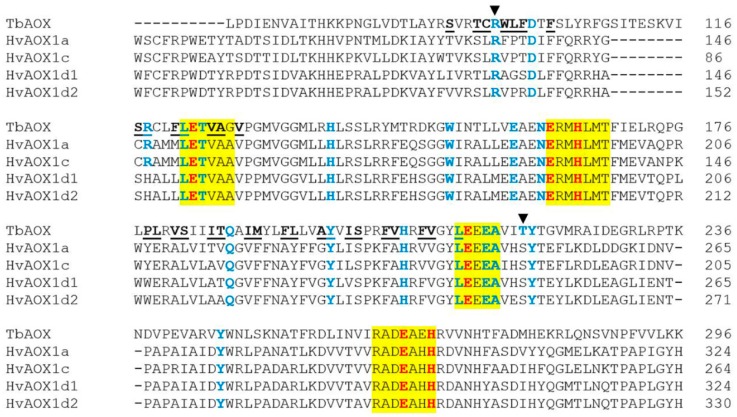
Protein alignment of HvAOX isoforms with *T. brucei* AOX (TbAOX). Color scheme follows that previously established by Brew-Appiah et al. [12]: residues highlighted in yellow indicate conserved motifs. The residues bolded in red are amino acids proposed to coordinate the diiron center of the active site. Residues bolded in blue have been experimentally tested for loss of activity by previous researchers. Underlined and bolded residues are involved in the TbAOX hydrophobic cavity. The dark arrows indicate the residues R96 and T219.

**Figure 2:**
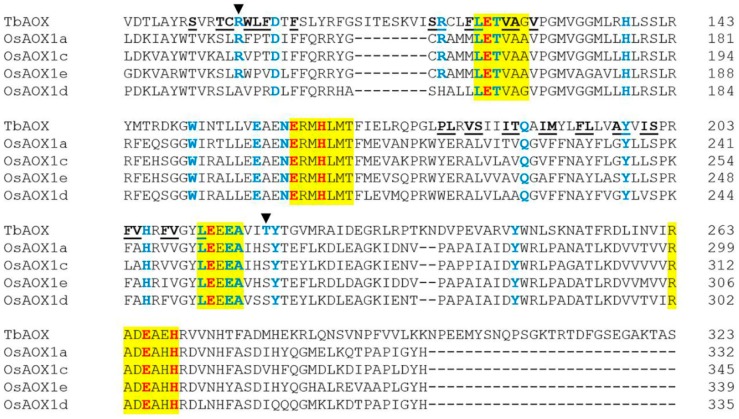
Protein alignment of OsAOX isoforms with *T. brucei* AOX (TbAOX). Color scheme follows that previously established by Brew-Appiah et al. [12]: residues highlighted in yellow indicate conserved motifs. The residues bolded in red are amino acids proposed to coordinate the diiron center of the active site. Residues bolded in blue have been experimentally tested for loss of activity by previous researchers. Underlined and bolded residues are involved in the TbAOX hydrophobic cavity. The dark arrows indicate the residues R96 and T219.

The crystal structure of *Trypanosoma brucei* AOX (TbAOX) indicates that it is a diiron carboxylate protein with the diiron core ligated by four conserved glutamate residues (E123, E162, E213, E266) and two histidine residues (H165, H269) [24,25]. This evolutionary feature has been further confirmed by other researchers who have shown that these six residues can, in most cases, be found in four AOX-indicative motifs in many plant species [26]. Mutation of any one of these six residues is extremely detrimental to AOX activity. In addition, research has shown that specific mutations in other critical residues partially or completely inactivate AOX [24,25,26,27,28,29]. Overall, these important residues are conserved between TbAOX, wheat AOX, HvAOX, and OsAOX isoforms, underscoring their relevance across species (Table S2), [12]. One notable exception is in wild and domesticated wheat, where isoforms have been found that are missing one of the AOX-indicative motifs and, therefore, lack some of the six residues found to be crucial for the active site. These wheat isoforms were dubbed “AOX-like” [12]. It is logical to therefore assume attenuated activity of these isoforms. However, if these isoforms have been evolutionarily sustained in the domesticated hexaploid species, they may have some yet unknown essential function. If these “AOX-like” isoforms are found in other monocots or other plant species in general, it may give clues as to how they have functionally evolved over time. Another exception is a T219S substitution conserved in wheat, barley, and rice (Figure 1 and Figure 2, and Table S2), [12]. In recombinant *T. brucei* AOX (rTbAOX), a T219V mutation resulting in a significant change in side chain chemistry (polar to nonpolar) and, possibly, enzyme configuration or domain features, causes an almost complete loss of function [27]. The conserved T219S substitution seen in the monocots above may have steric implications, due to the lost methyl group, and this may affect the three-dimensional structure of the protein and change functionality in ways that are currently unknown. There is also an R96A substitution in OsAOX1d which, in other species, causes a drastic loss of activity (Figure 2, Table S2) [28]. A deterioration of OsAOX1d efficiency is, therefore, a viable hypothesis. In hexaploid wheat, these two residues, R96 and T219, are absent in the “AOX-like” isoforms. Given that this has been discussed just within the monocots, it is possible that substitutions or deletions at these two locations may also be seen in other plant species. It would be worth knowing how these and other changes are definitive of a divergence between the parasitic lineage and other vascular and nonvascular plant forms. If some, or all of these changes are conserved in all plant forms, it is still plausible that AOX isoforms may function differently in the grasses when posttranslational modifications are considered [30,31,32,33,34,35,36]. Moreover, it has been theorized that mutations deleterious to an organism may be benign in others, and this may be the case with the monocots [25,37]. These observations call attention to the need for more functional characterization of the *AOX* gene families of other monocots, both wild and domesticated, to fully decipher which changes inhibit or enhance enzyme performance and how these alterations aid in the success or failure of a species in a unique environment.

## 4. Beyond the Diiron Center: Can Functionality Be Defined by the Hydrophobic Cavity?

The touted high efficiency of TbAOX [25] may be due to its function in a unicellular organism, which necessitates the optimization of genes and translated proteins, in order to compensate for a lack of genetic redundancy characteristic of multicellular organisms. Another domain recently emphasized as critical for AOX functionality is the hydrophobic cavity where the environment created by a mix of 33 polar and hydrophobic residues facilitates quinol binding in the TbAOX active site [25]. The discovery of this region provides an exceptional opportunity for plant researchers to look beyond the four critical AOX motifs already identified as influencers of enzyme activity [24,26]. A comparison of TbAOX and HvAOX isoforms reveals similarities and deviations from the parasitic isoform, as well as clade-dependent variations within the hydrophobic cavity (Figure 1, Table 2 and Table S3). Nine out of the 33 residues show complete conservation with TbAOX (F102, L122, V125, A126, V128, Y198, S201, V209, L212) signaling that these may be crucial for active site efficiency as they have been conserved between two unrelated species (Figure 1, Table S3). Notably, these same nine residues are conserved in the wheat isoforms with the exception of AOX-like isoforms [12]. The amino acid glycine, not present in the TbAOX hydrophobic cavity, is found in the same domain of wheat and HvAOX isoforms (Figure 1, Table 2 and Table S3, [12]. The evident clade-specific disparities and distributions of hydrophobic, polar, and cyclic residues within the HvAOX family may affect enzyme proficiency, and this is an avenue that needs to be elucidated via more molecular work. Although in some cases, the specific amino acids differ, the chemistry of the side chains may be similar across species (Table 2 and Table S3). One can, therefore, explore how such substitutions with conserved chemistry but divergent sterics in the active site, may modify AOX function in plants.

In wheat and barley, there are eight substitutions which are conserved in this hydrophobic cavity (L179E, V181A, S182L, I189V, M190F, F193A, L194Y, V205A) (except in some wheat AOX-like isoforms), again showing the interrelatedness of the two monocot species (Table 2 and Table S3), [12]. In addition, in barley, similar to wheat (with the exception of some AOX-like isoforms), there are five residues or substitutions conserved in the hydrophobic cavity of the HvAOX1d isoforms (S117, R118H, F121L, P178W, F208) (Table 2 and Table S3, [12]). In rTbAOX, the mutations R118A and R118Q cause a severe loss of activity despite chemistry retention in the latter mutation [27]. In R118H, the polarity is conserved despite the substitution, however the difference is a transition to a cyclic amino acid. Depending on the structural or functional context, the difference in size may have notable effects. The R118H substitution found in barley and wheat therefore opens up another avenue of inquiry into the evolution of AOX function in monocot species. HvAOX1d1 and HvAOX1d2 diverged with the wheat AOX1d Group 1 and Group 2 clades, respectively [12]. It is noteworthy that for AOX1d Group 2, the hydrophobic cavity residues of these related monocots are identical in both chemistry and sterics (Table 2 and Table S3). This gives the opportunity to study this domain in the context of the AOX1d Group 2 clade and, potentially, extrapolate the effects seen in barley mutational studies to wheat. This may be an acceptable option in cases where researchers have easier access to barley varieties than to diploid wheat germplasm, due to material transfer complications or undefined growth habits of wild species, which may set back a researcher’s time table. Also, in some cases, there may be the issue of the inadequacy of seed produced by the wild diploid species which may hamper the range of experiments one is able to perform. It may be more expedient to use domesticated models, like barley, in some of these analyses. In addition, there are other residues or substitutions peculiar to AOX1d Group 1 (S91I, T94, L98G, F99S, T186) or AOX1d Group 2 (S91V, F99R, T186A) that are conserved between the AOX1d subclades of bread wheat and barley (Table 2 and Table S3, [12]) further highlighting the close relationship in this gene family between the two species. Given the clade-specific similarities between barley and its close polyploid relative, mutational studies may lead to applicable conclusions in both crops.

Nonetheless, there are also differences between the wheat and barley AOX isoforms in the hydrophobic cavity. Excluding the wheat “AOX-like” isoform, where there is an I200V change as well as absent amino acids, the residues of HvAOX1a are identical to the same clade in wheat, except for I185, where a hydrophobic residue is replaced by a polar one (HvAOX1a: I185T) (Table 2 and Table S3). The hydrophobic, polar, and cyclic residues in HvAOX1c are identical to the counterpart clade in wheat with a single deviation at I200, where there is an isoleucine or valine in wheat and a leucine in barley (HvAOX1c: I200L) (Table 2 and Table S3). The AOX1c clade is absent in the ancestral diploid wheat *Aegilops tauschii*, while present in barley (Table 2 and Table S3), [12]. Another significant area where the wheat and barley species differ is in the presence of wheat “AOX-like” isoforms which highlight the effect polyploidy can have on neo- or subfunctionalization. In addition to missing some of the six highly conserved residues in the diiron center, these “AOX-like” proteins lack several residues in the hydrophobic cavity (Table 2 and Table S3). The effect of these changes, in comparison to the HvAOX proteins, is yet to be studied, and leads to several hypotheses; these wheat proteins could be less efficient than non-like isoforms in the same clade. On the other hand, these “AOX-like” proteins in wheat may have evolved high functionality in the same role, a possibility seen in other proteins, or in novel roles separate from the hydrophobic cavity [38,39,40]. These “AOX-like” proteins missing critical residues which potentially impair function may, in fact, be utilized in the same way as other plant proteins known as “limping enzymes”, where the loss of catalytic activity coupled with the retention of other functions allows for noncanonical specialization [41]. One example is the chitinase-like wheat seed protein XIP-I, which lacks enzymatic activity and, instead, works as a competitive inhibitor of xylanases and amylases ([41] and references therein). Two other chitinase-like proteins, CTL1 and CTL2, have no hydrolytic activity, but are expressed during cell wall thickening, and are thought to be important in the interaction between cellulose microfibrils and hemicellulose ([41] and references therein). There is a wealth of literature to show that these limping enzymes are pervasive in the plant world and their characterization is ongoing ([41] and references therein). Given the close relationship between the AOX proteins in wheat and barley, as well as the apparent presence of putative “limping” AOX isoforms in the former, studies involving the residues in this critical hydrophobic domain are imperative in the elucidation of AOX functionality in the grasses and plants, in general.

Mirroring the observations in barley and wheat, the same nine residues in the hydrophobic cavities of OsAOX isoforms show complete conservation with TbAOX (F102, L122, V125, A126, V128, Y198, S201, V209, L212) highlighting their possible importance in function (Figure 2, Table S3, [12]). In rice, there are ten substitutions conserved in all the OsAOX isoforms (S91T, C95L, L98P, L179E, V181A, S182L, M190F, F193A, L194Y, V205A) (Figure 2, Table 2 and Table S3), and some of these substitutions are identical to those in wheat and barley (L179E, V181A, S182L, M190F, F193A, L194Y, V205A) (Table 2 and Table S3). This shows the interrelatedness of these grass species, despite the earlier divergence of the rice AOX isoforms [12]. OsAOX1a shares the I185T substitution with the same clade in barley, which deviates from the I185A change in wheat (Table 2 and Table S3). In OsAOX1c, there are three points of deviation from wheat (T94A, I200L, and F204L) (Table 2 and Table S3). This highlights differences achieved by earlier divergence of the rice AOX1c clade with respect to wheat and barley in the hydrophobic cavity [12]. Another observed contrast in wheat and barley, when compared to rice, is the substitution I189V, which is conserved in all wheat and barley AOX isoforms, but not in rice (OsAOX1e: I189A) (Table 2 and Table S3).

Although absent in barley, the AOX1e clade is found in rice, and the residue W97 is conserved in the hydrophobic cavities of both TbAOX and the AOX1e clades of wheat and rice (Table 2 and Table S3). None of the other residues (T94A, C95I/M, A197T, I200A, F204V, F208M) previously reported as unique to the wheat AOX1e clade were found in rice (Table 2 and Table S3, [12]). However, in some of these cases, even though the substitution is not conserved, the chemistry is. For example, in wheat, the A197T substitution is A197S in OsAOX1e, maintaining the polar chemistry at this site. The I200A is I200L in OsAOX1e, conserving the hydrophobic property. In both cases, functionality of this clade may vary based on steric properties introduced by the side chain bulk. Due to the absence of the AOX1e clade in barley, rice may be exploited as a diploid model for functionality in this clade, along with the *Ae. tauschii* AOX1e isoform (AetAOX1e). While AetAOX1e maintains a lot of the unique residues found in the hexaploid counterpart, once again, there may be issues with growth habits, low seed production or material transfer agreements that may make characterization of the rice *AOX1e* gene and protein isoform a more convenient alternative in some geographic regions. On the other hand, the availability of both diploid wheat and rice *AOX1e* genes and translated isoforms allows for the exploration of gene expression variations, as well as the elucidation of the effect of specific residue changes on the enzyme efficiency of this clade during the evolution of the grasses.

Much like in wheat and barley, the same five residues or substitutions are conserved in the hydrophobic cavity of OsAOX1d (S117, R118H, F121L, P178W, F208) (Table 2 and Table S3). The similarities in these three species in the grass (Poaceae) family may suggest similar functionality of these isoforms and highlight the importance of these residues and substitutions in grasses. OsAOX1d precedes the divergence of the wheat and barley AOX1d isoforms into Group 1 and Group 2, and it may be worth clarifying how this difference affects the functionality of this isoform between these species [12]. It must be noted that OsAOX1d has two of the same three substitutions unique to the wheat AOX1d Group 2 (F99R, T186A), while all the other residues of OsAOX1d found in regions where the wheat and barley are unique, mirror other residues or substitutions in other clades (Table 2 and Table S3). This may present an opportunity to use the rice OsAOX1d as a built-in mutation template to explore how specific substitutions between the AOX1d clade and other clades change functionality between these two clades. The similarities exclusive to wheat and rice, or exclusive to wheat and barley, brings to the fore the question of model suitability. If the aim is to study the clade-specific gene expression and regulation, it may be constructive to utilize a diploid monocot with a similar clade and, then possibly use the results to inform work done on more complex polyploid systems. If the goal is to analyze enzyme function and kinetics, one could utilize a heterologous system for the investigation of mutants obtained via site-directed mutagenesis [15,30,42]. It may be helpful to first mutate the residues known to be conserved between all the monocots and TbAOX. Out of these nine, there are mutation studies on three of them (L122, Y198, L212) all of which have shown over 50% loss of activity [24,28,29]. There is, therefore, an opportunity to investigate the other six conserved residues. One could then target those residues that have been shown to be clade-specific. In the era of genome editing, one could also modify these residues *in planta*, in order to show how the variations made affect growth, development, and response to stress [43].

## 5. Exploration of the Dimer Interface in Monocot AOX Isoforms

The TbAOX crystal structure reveals a dimerization domain with six completely conserved residues and 12 semi-conserved residues [27]. Some of these residues lead to significant loss of activity when mutated (H138, Q187) [24]. The six completely conserved residues are maintained in wheat (exception is the “AOX-like” isoform), barley, and rice AOX isoforms (Table 3). This indicates that these proteins most likely also exist as dimers, an observation previously made by other researchers [14]. With regards to the 12 semi-conserved residues for the dimer interface, six are identical to TbAOX (M131, L139, S141, A159, M167, R180) in all five monocots (except where absent in the “AOX-like” isoform in wheat), suggesting the importance of these residues for functionality across species. Three other substitutions (M145F, D148S, I183V) are also conserved in all five monocots (with two exceptions in wheat, where it is absent or has a D148N in two of three “AOX-like” isoforms) (Table 3). In M145F, a change from a hydrophobic to a cyclic residue may have steric implications that could affect how the dimerization domain contributes to enzyme functionality and efficiency. In D148S, the chemistry of the sidechain is conserved (polar), however, the substitution leads to the loss of charge in this location, and it is unknown how this may change enzyme activity. In I183V, although the chemistry is conserved, there is a change in side chain bulk which may have steric implications in the active site.

There are similarities and differences between the five species in the dimerization domain. The substitution M135A is consistent in the three grasses which have the AOX1e clade (Table 3). Three substitutions in the AOX1d clade are conserved between wheat and barley in the dimerization domain (M135V, R147H, L156M) (Table 3). At these same three locations, OsAOX1d deviates from the four triticoid AOX1d clades; M135 and L156 are conserved with TbAOX, while there is a R147Q substitution which maintains the sidechain chemistry at the location, albeit with a loss of charge. This R147Q substitution is found in the Type 1 AOX clades (AOX1a/c/e) clades as well, but not in “AOX-like” isoforms (Table 3). Another region that is involved in dimerization is characterized by three cysteine residues, the first two (Cys I, CysII) of which, in *A. thaliana*, are involved in the formation of a disulfide bond leading to an inactive dimer [44]. When this bond is reduced, AOX is activated [44]. In *A. thaliana*, it has been shown that substitutions at these three positions can change the AOX isoform response to metabolite activators, such as succinate, pyruvate, and glyoxylate [30]. These three cysteines, which have been analyzed in barley and rice, were compared to the AOX isoforms from wheat [12,14]. In the AOX1a and 1c clades, the major pattern is CCL. The exceptions are the AOX-like isoforms, as well as the diploid wheat isoforms (Table 4, Figure S1). The presence of the first two cysteines may, therefore, indicate inactive dimer in vivo, due to the presence of the cysteines for the disulfide bridge. In the isoforms missing these two cysteines, it may be that these isoforms are constantly active, as has been suggested in barley [14]. In rice AOX1d, as well as wheat AOX1d group 1, the pattern is SSL, while barley shares the CSL pattern with wheat AOX1d group 2 (Table 4, Figure S1). These observations may indicate that the dimers in these clades cannot be inactivated. It is difficult to predict the response of these isoforms to the metabolites mentioned, as it has been shown that substitutions and the subsequent effect on response differs between the clades [30]. There is an opportunity here for researchers to determine how the triad of cysteines affects monocots and whether the same metabolites can be used to activate these isoforms. The high conservation between barley and bread wheat AOX may allow the utilization of the outcomes of barley research in the study of the more complex polyploid.

To summarize, the conservations and substitutions observed give rise to diversity in sidechain chemistry, as well as changes in sterics, due to the size of the substituted amino acid. These modifications require careful consideration, in order to determine what the effects on enzyme dimerization and efficiency in the context of both development and stress tolerance. Some of the diversity observed at both the dimerization interface (Table 3) and within the cysteine triad (Table 4, Figure S1) may lead to stronger or weaker homodimerization, which may affect enzyme activation and function. A question worth answering is whether similar metabolites activate AOX in the cereals and monocots in general. The possibility of the induction of monocot AOX isoforms by additional metabolites is also an enticing prospect. Another interesting question that arises is whether weaker homodimerization allows for heterodimerization of the monomer with another protein, thereby giving rise to new functionality in plants. This may be a question best answered using molecular studies with the wheat “AOX-like” isoforms. One approach would be to create and overexpress AOX-reporter fusions, in planta, under various conditions. An antibody targeting the reporter can then be used to pull down the gene–reporter fusion, as well as any other proteins associated with this chimeric protein. Interactions between each associated protein and the AOX-reporter fusion could then be studied further [45,46,47].

## 6. Conclusions

Previous researchers have shown the importance of AOX in stress tolerance, which in the case of grasses, could help boost food production. Between studies on wheat, barley, and rice, we can see possibilities provided by the presence of positive and negative regulators, as well as specific motifs, which may help to determine the spatiotemporal regulation of the *AOX* gene family in grasses. In addition to the work done by earlier researchers emphasizing how certain residue configurations may potentially lead to higher enzyme efficiencies [12,14,25], we show, here, that clade similarities and differences within and between wheat, barley, and rice in the hydrophobic cavity and dimer interface provide additional support for the theory of clade-dependent functionality, and show ways in which specific domains may allow for distinct structural possibilities in the efficiency of this protein family in the grasses and, possibly, in other plant species. The picture emerging challenges and encourages us to broaden our definition of model organism to include species which may, under normal circumstances, not qualify for a particular subject area or geographic region, or fulfill all the physiological requirements previously attributed to model organisms. Another insightful area of research may look at how *AOX* gene families in multicellular plants have evolved under various selective pressures, and how these adaptations have contributed to not only a resistance to environmental stresses but, also, to the robust growth of the species. This idea has already shown promise in the context of the utilization of TbAOX, which is active at the human body temperature, in the treatment of debilitating human conditions [25]. An examination of the contribution of specific AOX isoforms to the domination of monocot species in a distinct environment may be worthwhile as has been demonstrated with the reproductive success of thermogenic plants [48]. One question that arises is how does MRR involving responses of diverse AOX isoforms function in the context of these clades? Another is, are clade-specific genes expressed in a tissue-specific manner in some or all monocots? One such monocot which needs more study is maize, the last of the quartet of monocot species responsible for a significant portion of food production [1]. While there has been some work done on *AOX* genes in maize [49,50,51,52,53,54], the new information discovered should hopefully spur a more extensive characterization of the gene and protein family to better understand its evolution and, hopefully, utilize this information in food production. Another monocot which needs study is *Panicum virgatum*, which, while not used for food, has immense potential in the field of sustainable bioenergy production, where *AOX* has also been shown to increase biomass [55,56,57]. There is the hope that the information garnered about the *AOX1e* clade in wheat and rice may aid in research of this gene family in *P. virgatum*, which also contains an *AOX1e* gene [12]. It is worth mentioning that there is an abundance of germplasm available for wheat, barley, and rice, and it is entirely possible that some cultivars or ancestral relatives may be shown to have clades that are absent in the respective reference genomes. Depending on the expression patterns and positive contributions made by certain clades, there is an opportunity for the introduction of beneficial clades into marketable cultivars. Taken together, the discoveries made by previous researchers in wheat, barley, and rice open up new avenues for future studies to further our scientific understanding of the AOX protein family from diverse evolutionary origins, and leverage the information in the quest for both plant molecular and evolutionary characterization, as well as global food security.

## Figures and Tables

**Table 1:** Summary of binding sites of known positive and negative regulators of *AOX* found in barley (*HvAOX*) and rice (*OsAOX*) promoters (−1 to 1500 bp upstream of the ATG start site).

	*HvAOX1a*	*HvAOX1c*	*HvAOX1d1*	*HvAOX1d2*	*OsAOX1a*	*OsAOX1c*	*OsAOX1e*	*OsAOX1d*
ANAC013	3	1	-	-	3	-	-	-
ANAC017	2	1	-	-	2	-	-	-
AtWRKY63	-	-	-	1	-	-	-	-
ANAC053	2	-	-	-	2	-	-	-
ANAC078	1	-	-	-	-	-	1	-
ABI4	3	-	4	3	-	-	-	-
CTTGNNNNNCAMG	2	2	-	-	2	-	-	-
YTTGNNNNNVAMV	4	2	1	2	6	2	1	2

**Table 2:** A comparison of 24 residues in the hydrophobic cavity of TbAOX, hexaploid wheat AOX (TaAOX), *Triticum urartu* AOX (TuAOX, wild diploid wheat ancestor), *Aegilops tauschii* AOX (AetAOX, wild diploid wheat ancestor), HvAOX, and OsAOX. The nine residues common amongst all the protein isoforms are excluded. The wheat residues are from Brew-Appiah et al. [12], and the barley and rice residues are from Wanniarachchi et al. [14]. dG1 and dG2 refer to AOX1d Group 1 and AOX1d Group 2 respectively. * Indicates diploid wheat isoforms.

	AOX Isoforms	TbAOX Residues and Positions in the Hydrophobic Cavity																							
		S	T	C	W	L	F	S	R	F	P	L	V	S	I	T	I	M	F	L	A	I	F	V	F
		91	94	95	97	98	99	117	118	121	178	179	181	182	185	186	189	190	193	194	197	200	204	205	208
	HvAOX1a	T	S	L	F	P	T	C	R	M	Y	E	A	L	T	V	V	F	A	Y	G	I	F	A	V
	TaAOX1a-2AL	T	S	L	F	P	T	C	R	M	Y	E	A	L	A	V	V	F	A	Y	G	I	F	A	V
	TaAOX1a-2BL	T	S	L	F	P	T	C	R	M	Y	E	A	L	A	V	V	F	A	Y	G	I	F	A	V
	TaAOX1a-2DL	T	S	L	F	P	T	C	R	M	Y	E	A	L	A	V	V	F	A	Y	G	I	F	A	V
	TaAOX1a-like-2DL	-	-	-	-	-	-	-	-	-	Y	E	A	L	A	V	V	F	A	Y	G	V	F	A	V
	TuAOX1a *	T	S	L	F	P	T	C	R	M	Y	E	A	L	A	V	V	F	A	Y	G	I	F	A	V
	AetAOX1a *	T	S	L	F	P	T	C	R	M	Y	E	A	L	A	V	V	F	A	Y	G	I	F	A	V
	OsAOX1a	T	S	L	F	P	T	C	R	M	Y	E	A	L	T	V	V	F	A	Y	G	L	F	A	V
	HvAOX1c	T	S	L	V	P	T	C	R	M	Y	E	A	L	A	V	V	F	A	Y	G	L	F	A	V
	TaAOX1c-6AL	T	S	L	V	P	T	C	R	M	Y	E	A	L	A	V	V	F	A	Y	G	I	F	A	V
	TaAOX1c-6BL	T	S	L	V	P	T	C	R	M	Y	E	A	L	A	V	V	F	A	Y	G	V	F	A	V
	TaAOX1c-6DL	T	S	L	V	P	T	C	R	M	Y	E	A	L	A	V	V	F	A	Y	G	V	F	A	V
	TuAOX1c *	T	S	L	V	P	T	C	R	M	Y	E	A	L	A	V	V	F	A	Y	G	V	F	A	V
	OsAOX1c	T	A	L	V	P	T	C	R	M	Y	E	A	L	A	V	V	F	A	Y	G	L	L	A	V
	Put.TaAOX1e-3DS	T	A	I	W	P	T	C	R	M	Y	E	A	L	V	V	V	F	A	Y	T	A	V	A	M
	AetAOX1e *	T	A	M	W	P	T	C	R	M	Y	E	A	L	A	V	V	F	A	Y	T	A	V	A	M
	OsAOX1e	T	S	L	W	P	V	C	R	M	Y	E	A	L	A	V	A	F	A	Y	S	L	F	A	I
	OsAOX1d	T	S	L	V	P	R	S	H	L	W	E	A	L	A	A	V	F	A	Y	G	V	F	A	F
**dG1**	HvAOX1d1	I	T	L	A	G	S	S	H	L	W	E	A	L	A	T	V	F	A	Y	G	V	F	A	F
	TaAOX1d-2AL.1	I	T	L	A	G	S	S	H	L	W	E	A	L	A	T	V	F	A	Y	G	V	F	A	F
	put.TaAOX1d-like-4AS	-	-	-	-	-	-	S	H	L	C	E	A	L	P	T	V	F	A	Y	G	V	F	A	F
	TuAOX1d.2 *	I	T	L	K	G	S	S	H	L	W	E	A	L	A	T	V	F	A	Y	G	V	F	A	F
	AetAOX1d-like *	I	T	L	A	G	S	S	H	L	-	-	-	-	-	-	V	F	A	Y	G	I	L	-	-
**dG2**	HvAOX1d2	V	S	L	V	P	R	S	H	L	W	E	A	L	A	A	V	F	A	Y	G	I	F	A	F
	TaAOX1d-2AL.2	V	S	L	V	P	R	S	H	L	W	E	A	L	A	A	V	F	A	Y	G	I	F	A	F
	TaAOX1d-2DL	V	S	L	V	P	R	S	H	L	W	E	A	L	A	A	V	F	A	Y	G	I	F	A	F
	TuAOX1d.1 *	V	S	L	V	P	R	S	H	L	W	E	A	L	A	A	V	F	A	Y	G	I	F	A	F
	AetAOX1d *	V	S	L	V	P	R	S	H	L	W	E	A	L	A	A	V	F	A	Y	G	I	F	A	F

**Table 3:** A comparison of residues in the dimerization interface of the TbAOX, hexaploid wheat AOX (TaAOX), *Triticum urartu* AOX (TuAOX, wild diploid wheat ancestor), *Aegilops tauschii* AOX (AetAOX, wild diploid wheat ancestor), HvAOX, and OsAOX. The wheat residues are from Brew-Appiah et al. [12], and the barley and rice residues are from Wanniarachchi et al. [14]. dG1 and dG2 refer to AOX1d Group 1 and AOX1d Group 2, respectively. * Indicates diploid wheat isoforms.

	AOX Isoforms	Completely Conserved with TbAOX						Semi-Conserved with TbAOX											
		H138	L142	R143	R163	L166	Q187	M131	M135	L139	S141	M145	R147	D148	L156	A159	M167	R180	I183
	HvAOX1a	H	L	R	R	L	Q	M	M	L	S	F	Q	S	L	A	M	R	V
	TaAOX1a-2AL	H	L	R	R	L	Q	M	M	L	S	F	Q	S	L	A	M	R	V
	TaAOX1a-2BL	H	L	R	R	L	Q	M	M	L	S	F	Q	S	L	A	M	R	V
	TaAOX1a-2DL	H	L	R	R	L	Q	M	M	L	S	F	Q	S	L	A	M	R	V
	TaAOX1a-like-2DL	H	L	R	R	L	Q	M	V	L	S	F	H	S	M	A	M	R	V
	TuAOX1a *	H	L	R	R	L	Q	M	M	L	S	F	Q	S	L	A	M	R	V
	AetAOX1a *	H	L	R	R	L	Q	M	M	L	S	F	Q	S	L	A	M	R	V
	OsAOX1a	H	L	R	R	L	Q	M	M	L	S	F	Q	S	L	A	M	R	V
	HvAOX1c	H	L	R	R	L	Q	M	M	L	S	F	Q	S	L	A	M	R	V
	TaAOX1c-6AL	H	L	R	R	L	Q	M	M	L	S	F	Q	S	L	A	M	R	V
	TaAOX1c-6BL	H	L	R	R	L	Q	M	M	L	S	F	Q	S	L	A	M	R	V
	TaAOX1c-6DL	H	L	R	R	L	Q	M	M	L	S	F	Q	S	L	A	M	R	V
	TuAOX1c *	H	L	R	R	L	Q	M	M	L	S	F	Q	S	L	A	M	R	V
	OsAOX1c	H	L	R	R	L	Q	M	M	L	S	F	H	S	L	A	M	R	V
	put.TaAOX1e-3DS	H	L	R	R	L	Q	M	A	L	S	F	Q	S	L	A	M	R	V
	AetAOX1e *	H	L	R	R	L	Q	M	A	L	S	F	Q	S	L	A	M	R	V
	OsAOX1e	H	L	R	R	L	Q	M	A	L	S	F	H	S	L	A	M	R	V
	OsAOX1d	H	L	R	R	L	Q	M	M	L	S	F	Q	S	L	A	M	R	V
dG1	HvAOX1d1	H	L	R	R	L	Q	M	V	L	S	F	H	S	M	A	M	R	V
	TaAOX1d-2AL.1	H	L	R	R	L	Q	M	V	L	S	F	H	S	M	A	M	R	V
	put.TaAOX1d-like-4AS	H	L	R	R	L	Q	M	V	L	S	F	H	N	M	A	M	R	V
	TuAOX1d.2 *	H	L	R	R	L	Q	M	V	L	S	F	H	S	M	A	M	R	V
	AetAOX1d-like *	H	L	R	R	L	-	M	V	L	S	F	H	S	M	A	M	-	-
dG2	HvAOX1d2	H	L	R	R	L	Q	M	V	L	S	F	H	S	M	A	M	R	V
	TaAOX1d-2AL.2	H	L	R	R	L	Q	M	V	L	S	F	H	S	M	A	M	R	V
	TaAOX1d-2DL	H	L	R	R	L	Q	M	V	L	S	F	H	S	M	A	M	R	V
	TuAOX1d.1 *	H	L	R	R	L	Q	M	V	L	S	F	H	S	M	A	M	R	V
	AetAOX1d *	H	L	R	R	L	Q	M	V	L	S	F	H	S	M	A	M	R	V

**Table 4:** Summary of the residues involved in the cysteine triad are known to determine in vivo dimer activation status, as well as responses to metabolites. Cys I and CysII are important for disulfide bond formation and dimer inactivation. The wheat residues are from Brew-Appiah et al. [12], and the barley and rice residues are from Wanniarachchi et al. [14]. dG1 and dG2 refer to AOX1d Group 1 and AOX1d Group 2 respectively. * Indicates diploid wheat isoforms.

	AOX Isoforms	Critical Cysteines			Putative Dimer Status In Vivo
		CysI	CysII	CysIII	
	HvAOX1a	C	C	L	Inactive
	TaAOX1a-2AL	C	C	L	Inactive
	TaAOX1a-2BL	C	C	L	Inactive
	TaAOX1a-2DL	C	C	L	Inactive
	TaAOX1a-like-2DL	-	-	L	Active
	TuAOX1a *	-	C	L	Active
	AetAOX1a *	-	C	L	Active
	OsAOX1a	C	C	L	Inactive
	HvAOX1c	C	C	L	Inactive
	TaAOX1c-6AL	C	C	L	Inactive
	TaAOX1c-6BL	C	C	L	Inactive
	TaAOX1c-6DL	C	C	L	Inactive
	TuAOX1c *	E	C	L	Active
	OsAOX1c	C	C	L	Inactive
	put.TaAOX1e-3DS	C	C	L	Inactive
	AetAOX1e *	C	C	L	Inactive
	OsAOX1e	C	C	L	Inactive
	OsAOX1d	S	S	L	Active
**dG1**	HvAOX1d1	C	S	L	Active
	TaAOX1d-2AL.1	S	S	L	Active
	put.TaAOX1d-like-4AS	S	S	L	Active
	TuAOX1d.2 *	S	S	L	Active
	AetAOX1d-like *	S	S	L	Active
**dG2**	HvAOX1d2	C	S	L	Active
	TaAOX1d-2AL.2	C	S	L	Active
	TaAOX1d-2DL	C	S	L	Active
	TuAOX1d.1 *	C	S	L	Active
	AetAOX1d *	C	S	L	Active

## Supplementary Materials

The following are available online at http://www.mdpi.com/1422-0067/19/10/2972/s1. Table S1: Summary of binding sites found for AOX positive and negative regulators in barley and rice. * Indicates sequences with one nucleotide deviation from the MDM. The location of nucleotide deviation is in bold and underlined, Table S2: Summary of residues of barley and rice AOX isoforms compared to *T. brucei* AOX residues that show effect on enzyme efficiency when mutagenized. The *T. brucei* AOX residues are in bold. * Indicates residues from Young et al. [28] and Crichton et al. [29]. ** Indicates residues Shiba et al. [27]. The rest of the residues are from Moore et al. [24], Table S3: A comparison of 33 residues in the hydrophobic cavity of TbAOX, hexaploid wheat AOX (TaAOX), *Triticum urartu* AOX (TuAOX, wild diploid wheat ancestor), *Aegilops tauschii* AOX (AetAOX, wild diploid wheat ancestor), HvAOX, and OsAOX. The wheat residues are from Brew-Appiah et al. [12] and the barley and rice residues are from Wanniarachchi et al. [14]. dG1 and dG2 refer to AOX1d Group 1 and AOX1d Group 2 respectively. * Indicates diploid wheat isoforms, Figure S1: Alignment of the diploid (TuAOX, AetAOX) and hexaploid wheat AOX (TaAOX) isoforms used in the location of Cys I (red box), CysII (blue box) and CysIII (green box).
