# Supplementary figures and images for: Considerations of AOX Functionality Revealed by Critical Motifs and Unique Domains
Source: Int J Mol Sci. 2018 Sep 29;19(10):2972. doi: 10.3390/ijms19102972 (PMC6213860; doi:10.3390/ijms19102972)

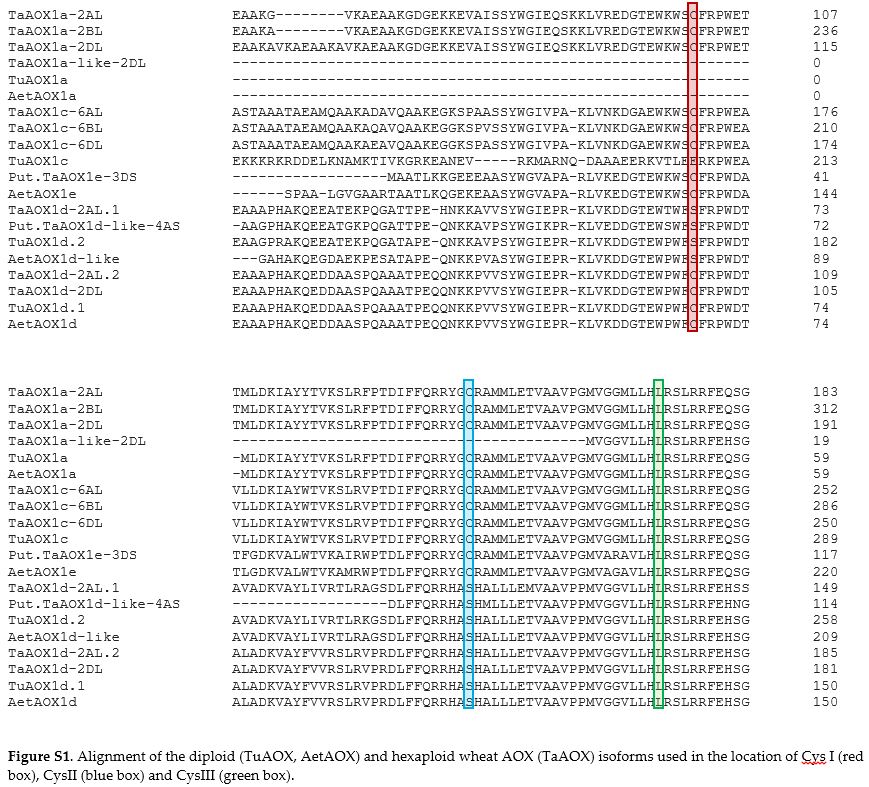

Supplement: Supplementary file 1 [file ijms-19-02972-s001.zip › Figure S1.JPG]
